# A novel gait analysis system for detecting abnormal hemiparetic gait patterns during robot-assisted gait training: A criterion validity study among healthy adults

**DOI:** 10.3389/fnbot.2022.1047376

**Published:** 2022-12-01

**Authors:** Daisuke Imoto, Satoshi Hirano, Masahiko Mukaino, Eiichi Saitoh, Yohei Otaka

**Affiliations:** ^1^Department of Rehabilitation, Fujita Health University Hospital, Toyoake, Aichi, Japan; ^2^Department of Rehabilitation Medicine I, School of Medicine, Fujita Health University, Toyoake, Aichi, Japan

**Keywords:** rehabilitation, gait analysis, robot-assisted gait training, validation study, cerebrovascular disorders

## Abstract

**Introduction:**

Robot-assisted gait training has been reported to improve gait in individuals with hemiparetic stroke. Ideally, the gait training program should be customized based on individuals’ gait characteristics and longitudinal changes. However, a gait robot that uses gait characteristics to provide individually tailored gait training has not been proposed. The new gait training robot, “Welwalk WW-2000,” permits modification of various parameters, such as time and load of mechanical assistance for a patient’s paralyzed leg. The robot is equipped with sensors and a markerless motion capture system to detect abnormal hemiparetic gait patterns during robot-assisted gait training. Thus, it can provide individually tailored gait training. This study aimed to investigate the criterion validity of the gait analysis system in the Welwalk WW-2000 in healthy adults.

**Materials and methods:**

Twelve healthy participants simulated nine abnormal gait patterns that were often manifested in individuals with hemiparetic stroke while wearing the robot. Each participant was instructed to perform a total of 36 gait trials, with four levels of severity for each abnormal gait pattern. Fifteen strides for each gait trial were recorded using the markerless motion capture system in the Welwalk WW-2000 and a marker-based three-dimensional (3D) motion analysis system. The abnormal gait pattern index was then calculated for each stride from both systems. The correlation of the index values between the two methods was evaluated using Spearman’s rank correlation coefficients for each gait pattern in each participant.

**Results:**

Using the participants’ index values for each abnormal gait pattern obtained using the two motion analysis methods, the median Spearman’s rank correlation coefficients ranged from 0.68 to 0.93, which corresponded to moderate to very high correlation.

**Conclusion:**

The gait analysis system in the Welwalk WW-2000 for real-time detection of abnormal gait patterns during robot-assisted gait training was suggested to be a valid method for assessing gait characteristics in individuals with hemiparetic stroke.

**Clinical trial registration:**

[https://jrct.niph.go.jp], identifier [jRCT 042190109].

## Introduction

Stroke is a serious and disabling disease worldwide (Feigin et al., 2014). Gait disorder is one of the main disabilities resulting from stroke (Jørgensen et al., 1995), leading to activity limitations and participation restrictions (Andrenelli et al., 2015). Gait training is the main treatment method to improve the gait ability of individuals with hemiparetic stroke (Jette et al., 2005; Latham et al., 2005), although more effective gait training methods are needed to obtain higher gait ability.

In recent years, robotic technology has been incorporated into gait training for individuals with hemiparetic stroke to improve individuals’ gait disorder. Robot-assisted gait training can provide intensive, repetitive, and task-oriented training for individuals with hemiparetic stroke who cannot walk independently by supporting their weight and movement partially or completely with a robotic control mechanism (Morone et al., 2017). Accumulating evidence has demonstrated the effectiveness of robot-assisted gait training among individuals with hemiparetic stroke (Cho et al., 2018; Mehrholz et al., 2020), and its use has been recommended in treatment guidelines (Calabrò et al., 2021).

However, gait training should ideally be individualized based on patients’ gait characteristics and longitudinal changes to maximize the effectiveness of the training. Appropriate assessment of gait characteristics in individuals with stroke can help plan treatment targets (Mulroy et al., 2003), monitor the effects of treatment (Toro et al., 2003), and predict the degree of improvement (Kaczmarczyk et al., 2012). A three-dimensional (3D) gait analysis system can objectively quantify gait characteristics and help in planning of treatment and evaluation of treatment effects (Baker et al., 2016). The gold standard method for quantitative gait analysis has been assessment of gait characteristics among individuals with hemiparetic stroke using marker-based motion capture systems in a special environment. However, these devices have not been widely used for clinical gait analysis due to several barriers, including equipment costs, installation and infrastructure, structured multifactorial gait assessment difficulties, and interpretation of a vast amount of complex gait data (Jang et al., 2017). Recently, markerless motion capture systems using a low-cost optical body tracking sensor have been proposed as an alternative to marker-based motion capture systems (Cerfoglio et al., 2022). Furthermore, gait analysis systems using small and lightweight wearable sensors such as inertial measurement units, pressure sensors, and acceleration sensors are revolutionizing gait assessment in research settings (Mohan et al., 2021). These devices have the potential to provide quantitative gait analysis in routine practice easily, even in the research phase. Thus, robot-assisted gait training combined with quantitative gait analysis may offer a new and powerful interventional tool for treating gait disorders in individuals with stroke. Although gait robots have the potential to obtain quantitative index values of gait characteristics based on information from the equipped sensors, to the best of our knowledge, a gait training robot that can provide individually tailored training based on gait characteristics measured using the robot during training has not been proposed.

We have developed a new gait training robot, Welwalk WW-2000 (WW-2000, Toyota Motor Corporation, Aichi, Japan), that permits modifications of various parameters, such as time and mechanical assistance load for a patient’s paralyzed leg. The robot is equipped with sensors and a markerless motion capture system to detect abnormal hemiparetic gait patterns during robot-assisted gait training (Nakashima et al., 2020). These functions enable appropriate robot settings while evaluating individuals’ gait characteristics; thus, the robot could provide individually tailored gait training. The validity of this system in assessing gait characteristics should be examined before the implementation and widespread use of this novel gait training robot in clinical settings. Therefore, this study aimed to investigate the criterion validity of the index values calculated by the gait analysis system of the robot among healthy adults.

## Materials and methods

### Participants

Twelve healthy adults without musculoskeletal disorders and neurological diseases participated in this study. This study protocol was approved by the Institutional Review Board of the Fujita Health University, Japan (IRB approval number: CR19-027) and was registered in the Japan Registry for Clinical Trials (jRCT 042190109) before study enrollment. All the participants provided written informed consent for the study.

### Instruments

**FIGURE 1:**
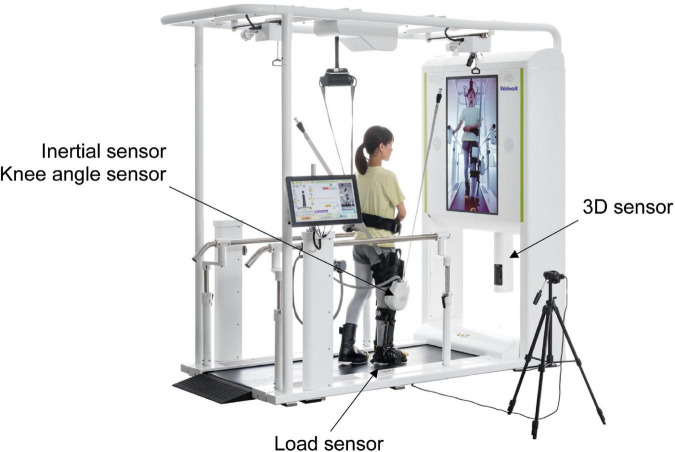
Overview of the Welwalk WW-2000. The three-dimensional (3D) sensor is placed below the front monitor at 0.6 m from the treadmill surface, and the distance between the sensor and object was 1.2 m. The 3D sensor, inertial sensor, and knee angle sensor are calibrated with the participants in an upright posture before walking. The load sensor is calibrated before the robot is attached to the participants.

The WW-2000 comprises a knee-ankle-foot robot, low floor treadmill, safety suspension device for body weight support, monitor for patient use, 3D sensor, and control panel. The robot detects a gait cycle using a load sensor located on the sole and monitors the knee joint angle with a knee angle sensor. Based on the data detected by the sensors, the robot uses the knee joint motor to assist the patient in flexion and extension of the knee joint during the swing and stance phases, respectively. A patient would place the robot on his or her paralyzed lower extremity and walk on the treadmill with the support of the robot. The WW-2000 is equipped with a gait analysis system to detect abnormal gait patterns during robot-assisted gait training. The components of the system include a 3D sensor (Xtion2, ASUS Japan Corporation, Tokyo, Japan) for markerless motion capture, inertial sensor, knee angle sensor, and load sensors (Figure 1). The WW-2000 was placed away from sunlight in the rehabilitation center at the Fujita Health University Hospital.

### Experimental tasks

The participants simulated nine abnormal gait patterns that were often manifested in individuals with hemiparetic stroke while wearing the robot (Mukaino et al., 2018). Each participant was instructed to walk with four grades of severity for each abnormal gait pattern, amounting to 36 gait trials. For each gait trial, the participants walked over 20 strides. Simulated abnormal gait patterns were as follows: hip hiking (Kerrigan et al., 2000), circumduction (Kerrigan et al., 2000), retropulsion of the hip (Davies, 2000), excessive hip external rotation (Perry and Schoneberger, 1992), excessive lateral shift of the trunk over the unaffected side (Perry and Schoneberger, 1992), knee extensor thrust (Perry and Schoneberger, 1992), medial whip (Menard et al., 1992), posterior trunk tilt (Perry and Schoneberger, 1992), and contralateral vaulting (Perry and Schoneberger, 1992). The treadmill speed was set at 0.55 km/h, and the participants were allowed to use the handrail during the assessment. Assistance in the knee joint extension motion in the stance phase and stepping in the swing phase was set to a minimum in the system. The robot leg controlled the participant’s knee joint flexion and extension movements during the swing phase. After sharing the definitions and reference movies of the abnormal gait patterns, the participants practiced to sufficiently simulate the abnormal gait patterns. The participants were asked to simulate four grades of the gait patterns, ranging from normal to most severe, with the same interval of severity between each grade (the movies during these tasks are included in Supplementary material). The measurements were performed after a physical therapist verified that all participants were able to adequately simulate the abnormal gait patterns.

### Data acquisition

**FIGURE 2:**
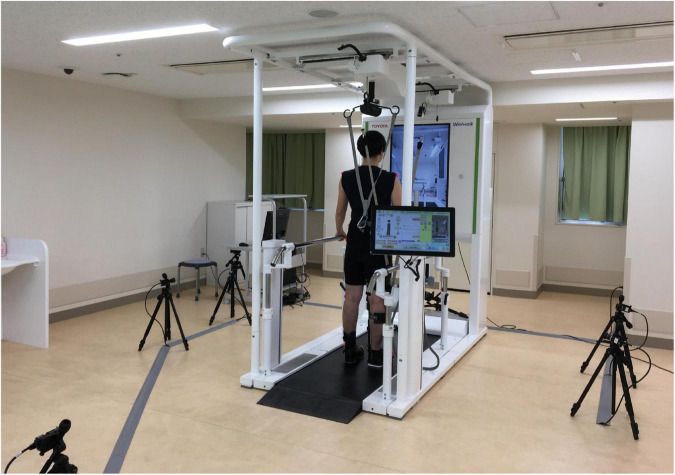
Measurement environment. Five CCD cameras were placed around the Welwalk WW-2000 to record gait patterns during the tasks using a marker-based motion capture system and the gait analysis system in Welwalk WW-2000.

The gait patterns during the tasks were recorded using the novel gait analysis system in the WW-2000 as well as a marker-based motion capture system (KinemaTracer^®^, Kissei Comtec Co., Ltd., Matsumoto, Nagano, Japan) (Figure 2). In the novel gait analysis system in the WW-2000, the 3D joint positions, lower limb tilt, and knee joint angle during the task were recorded using the 3D sensor, inertial sensor, and knee angle sensor, respectively, at a sampling frequency of 30 Hz. The 3D coordinates of the joint positions were estimated from the two-dimensional joint positions obtained using the skeletal tracking software (VisionPose^®^, NEXT-SYSTEM Co., Ltd., Fukuoka, Japan) combined with the depth information obtained from the 3D sensor. The estimated 3D joint positions were the bilateral shoulder, hip, knee, and ankle joints and midpoints of the bilateral shoulder and hip joints. The lower limb tilt and knee joint angle were detected by the inertial sensor (IMU–3 axis inertial sensor AU7684N1, TAMAGAWA SEIKI Co., Ltd., Nagano, Japan) located on the thigh and knee angle sensor located at the knee joint of the robot, respectively. The pitch and roll angles were calculated as the tilt of the lower limb using an algorithm that hybridized the gyro sensor and accelerometer signals built into the inertial sensor (TAMAGAWA SEIKI Co., Ltd., 2022). The load sensors located on the sole of the robot determined the stance phase of the gait cycle. The load sensor was calibrated before the participants wore the robot. The knee angle sensor was calibrated at the time when the participants stood up with the robot. The 3D and inertial sensors were calibrated in a static standing posture before gait.

In this study, we operated this system according to protocols described previously (Itoh et al., 2012; Matsuda et al., 2016; Tanikawa et al., 2016, 2021; Hishikawa et al., 2018). Briefly, the color markers with a diameter of 30 mm were placed on the participant’s body and robot, and the movements of these markers were recorded with five CCD cameras at a sampling frequency of 60 Hz. The color markers were placed on the following participant’s body parts: bilateral acromia iliac crests, hip joints (i.e., one-third of the distance from the greater trochanter on a line joining the anterior superior iliac spine and greater trochanter), toes (i.e., fifth metatarsal head), left knee joint (i.e., midpoint of the anteroposterior diameter of the lateral femoral epicondyle), and left ankle joint (i.e., lateral malleolus). In addition, color markers were placed on the covered parts of the robot. Markers were placed at three positions on the knee joint motor part of the robot to estimate the marker position and on the outside of the foot joint of the robot. The distances between the markers on the robot and participant’s body surface were measured using a tape. The positions of the knee and ankle joints were then estimated from the measured distance.

### Calculations of abnormal gait pattern index values

The index of gait abnormality was calculated for 15 strides, excluding the first two and last three steps of the recorded 20 strides. In the novel gait analysis in the WW-2000, the abnormal gait pattern index values were calculated according to the definitions shown in Table 1 using the information of the 3D joint positions, tilt of the robot, and knee joint angle of the robot recorded using the 3D sensor, inertial sensor, knee angle sensor, and load sensor, respectively. In the marker-based motion capture system, the abnormal gait pattern index values were calculated using the 3D positions estimated from the color markers following methods established in previous studies (Table 1; Itoh et al., 2012; Matsuda et al., 2016; Tanikawa et al., 2016, 2021; Hishikawa et al., 2018). Previous studies have confirmed the validity and reliability of the marker-based motion capture system in assessing gait pattern index values in healthy adults and in patients with hemiparetic stroke. These index values have been shown to correlate with the clinical severities of gait disorders in individuals with hemiparetic stroke assessed by observation or to differentiate between healthy adults and individuals with hemiparetic stroke (Itoh et al., 2012; Matsuda et al., 2016; Tanikawa et al., 2016, 2021; Hishikawa et al., 2018).

**TABLE 1:** Definitions of index values of abnormal gait patterns for the gait analysis system in the Welwalk WW-2000 and marker-based motion capture system.

Gait patterns	The gait analysis system using the Welwalk WW-2000	Marker-based motion capture system using three-dimensional gait analysis system
Hip hiking	The maximum displacement of the angles between the hip joint on the non-paralyzed side and paralyzed side during the swing phase.	The difference between the maximum value of the Z-coordinate of the hip joint marker during the swing phase and Z-coordinate of the contralateral hip joint marker at the same time, corrected for the mean left-right difference of the Z-coordinate during the double support phase.
Circumduction	The maximum amount of thigh angle displacement on the paralyzed side during the swing phase on the paralyzed side.	The difference in distance between the lateral most X-coordinate of the ankle joint marker during 25–75% of the swing phase and medial most X-coordinate during 25–75% of the stance phase.
Retropulsion of the hip	The position of the ankle joint relative to the hip joint on the paralyzed side at the end of the stance phase on the paralyzed side.	The mean distance between the Y-coordinates of the ankle and hip joints in the single stance phase.
Excessive hip external rotation	The maximum displacement of the external rotation angle on the paralyzed thigh during the swing phase on the paralyzed side.	The mean distance between the X-coordinates of the ankle joint and toe in the swing phase.
Excessive lateral shift of the trunk over the unaffected side	The maximum angle between the ankle and shoulder joints on the non-paralyzed side during the swing phase on the paralyzed side.	The mean distance between (1) the lateral most X-coordinate of the midpoint between the bilateral acromions in the part of the double stance phase in which the affected leg is located behind the unaffected leg and the swing phase of affected leg, and (2) the mean X-coordinate of the midpoint between the bilateral ankle joints in the part of the double stance phase in which the affected leg is located behind the unaffected leg.
Knee extensor thrust	The maximum extension speed on the paralyzed knee joint during the stance phase.	The difference between the maximum Y-coordinate velocity of the knee in the single stance phase of the affected leg and treadmill gait speed.
Medial whip	The maximum external rotation angle of the thigh on the paralyzed side from the late stance phase to the end of the next pre-swing phase.	The distance between the lateral most X-coordinate of the ankle joint during 75–100% of the stance phase and medial most X-coordinate of the ankle joint during 25–75% in the stance phase of the affected leg.
Trunk posterior tilt	The maximum angle formed by the line segments connecting the centers of the shoulder and hip joints during the standing posture and line segments connecting the centers of both shoulder and hip joints from the swing phase to the mid-stance phase on the paralyzed side.	θ1/θ2 × 100 where θ1 is the change in the angle between the vector joining the markers placed on the hip joint and iliac crest and vertical line in the coordinate space, and θ2 is the change in the angle between the vector joining the marker placed on the hip and knee joints and vertical line in the coordinate space in the part of the double stance phase in which the affected leg is located behind the unaffected leg and the swing phase of the affected leg.
Contralateral vaulting	The difference between the maximum value and the minimum value of the Z-coordinate of the hip joint of the non-paralyzed side during the swing phase on the paralyzed side.	The Z-coordinate value of the contralateral hip calculated as differences between mid-stance and mid-swing.

### Analysis

The median and interquartile range values of each abnormal gait pattern index were calculated using two methods for each of the four grades of severities. To examine the validity of the novel gait analysis in the WW-2000 against the maker-based motion analysis, the correlations of the index values between the two methods were evaluated using Spearman’s rank correlation coefficients for each gait pattern in each participant. The minimum, median, and maximum values of the correlation coefficient values for all the participants were then calculated for each gait pattern. We defined the strength of the correlation coefficient as follows: slight correlation, less than 0.20; low correlation, 0.20–0.39; moderate correlation, 0.40–0.69; high correlation, 0.70–0.89; very high correlation, more than 0.90 (Guilford, 1942; Kanda, 2013). All statistical analyses were performed using EZR (Saitama Medical Center, Jichi Medical University, Saitama, Japan), which is a graphical user interface for R (The R Foundation for Statistical Computing, Vienna, Austria) (Kanda, 2013).

## Results

Twelve healthy adults without musculoskeletal disorders or neurological diseases participated in this study. Of these, six were male. The mean (standard devotion) age, height, and weight of the participants were 27 (3) years, 165 (8) cm, and 56 (7) kg, respectively.

**FIGURE 3:**
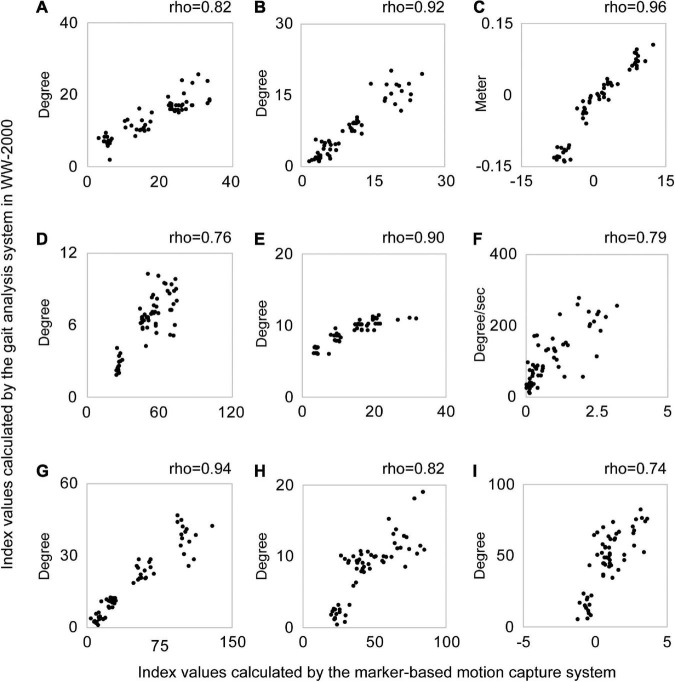
Scatter plots of the index values calculated by the gait analysis system in the Welwalk WW-2000 and marker-based motion capture system for each abnormal gait pattern in a typical case. Each graph is a scatter plot for each abnormal gait pattern index value calculated using the two methods in a typical case. Each abnormal gait pattern in the graph was as follows: **(A)** hip hiking, **(B)** circumduction, **(C)** retropulsion of the hip, **(D)** excessive hip external rotation, **(E)** excessive lateral shift of the trunk over the unaffected side, **(F)** knee extensor thrust, **(G)** medial whip, **(H)** posterior trunk tilt, and **(I)** contralateral vaulting. A plot in the graph shows the index value of the abnormal gait pattern calculated by the two systems at one stride. The horizontal axis shows the index values of the abnormal gait pattern calculated by the marker-based motion capture system and the vertical axis shows the index values calculated by the gait analysis system in the Welwalk WW-2000. Spearman’s rank correlation coefficient value (rho) of each abnormal gait pattern is shown above the graph. In this case, the abnormal gait pattern index values calculated by both systems confirm positive correlations between the two methods.

The scatter plots illustrating the relationship between the index values of the two methods for each abnormal gait pattern in an individual case are shown in Figure 3. The median index values calculated by the marker-based gait analysis system and Welwalk WW-2000 for each abnormal gait pattern are presented in Tables 2, 3, respectively. The index values of all abnormal gait patterns increased according to the severities in both methods. The median values of Spearman’s rank correlation coefficients for each abnormal gait pattern ranged from 0.68 to 0.93 (Table 4), indicating that the strength of correlation between the index values of the two methods for each abnormal gait pattern ranged from moderate to very high.

**TABLE 2:** Index values of abnormal gait patterns calculated by the marker-based motion capture system using three-dimensional (3D) gait analysis system.

Abnormal gait patterns	Grade 1	Grade 2	Grade 3	Grade 4
Hip hiking	5.91 (3.98)	16.56 (5.90)	22.00 (11.22)	30.72 (12.02)
Circumduction	2.22 (2.20)	6.82 (2.98)	11.13 (2.92)	19.58 (5.51)
Retropulsion of the hip	−9.18(4.75)	0.23 (8.63)	6.55 (6.66)	13.11 (7.91)
Excessive hip external rotation	26.39 (8.98)	51.38 (9.50)	68.35 (15.77)	81.25 (11.86)
Excessive lateral shift of the trunk over the unaffected side	4.24 (4.30)	13.67 (3.87)	20.16 (3.33)	26.67 (4.92)
Knee extensor thrust	0.15 (0.19)	0.71 (0.34)	1.23 (0.56)	2.25 (0.84)
Medial whip	11.94 (7.88)	34.43 (14.24)	54.48 (24.17)	88.05 (34.64)
Trunk posterior tilt	19.80 (13.36)	39.26 (17.39)	49.22 (15.18)	59.71 (16.46)
Contralateral vaulting	−0.54(1.17)	0.21 (2.68)	0.75 (2.48)	2.05 (4.23)

Data are presented as median (interquartile range) of the index values of abnormal gait patterns in all participants. These indexes are non-units as they are adjusted by the stride length.

**TABLE 3:** Index values of abnormal gait patterns calculated by the gait analysis system using the Welwalk WW-2000.

Abnormal gait patterns	Grade 1	Grade 2	Grade 3	Grade 4
Hip hiking, degree	8.16 (2.86)	12.71 (3.81)	16.03 (4.67)	20.72 (6.26)
Circumduction, degree	2.30 (2.34)	7.11 (3.95)	10.15 (4.71)	17.40 (6.47)
Retropulsion of the hip, meter	−0.16(0.05)	−0.05(0.10)	0.02 (0.07)	0.12 (0.09)
Excessive hip external rotation, degree	3.36 (2.02)	6.41 (2.12)	11.91 (5.80)	15.23 (5.74)
Excessive lateral shift of the trunk over the unaffected side, degree	6.01 (1.93)	8.75 (1.65)	10.15 (1.55)	11.61 (2.30)
Knee extensor thrust, degree/sec	20.00 (25.00)	78.44 (26.09)	127.19 (47.97)	223.44 (95.94)
Medial whip, degree	5.22 (3.29)	11.59 (5.17)	19.49 (7.45)	26.84 (13.13)
Trunk posterior tilt, degree	1.35 (4.71)	4.83 (5.10)	8.35 (4.37)	13.26 (4.71)
Contralateral vaulting, degree	11.92 (19.61)	27.17 (25.23)	33.69 (25.26)	52.32 (37.11)

Data are presented as median (interquartile range) of the index values of abnormal gait patterns in all participants.

**TABLE 4:** Spearman’s rank correlation coefficient values between the index values calculated by the gait analysis system in the Welwalk WW-2000 and the marker-based motion capture system.

Abnormal gait patterns	Spearman’s rho
Hip hiking	0.83 (0.69–0.92)
Circumduction	0.93 (0.80–0.95)
Retropulsion of the hip	0.92 (0.79–0.96)
Excessive hip external rotation	0.89 (0.76–0.93)
Excessive lateral shift of the trunk over the unaffected side	0.88 (0.70–0.93)
Knee extensor thrust	0.91 (0.61–0.95)
Medial whip	0.86 (0.76–0.96)
Trunk posterior tilt	0.68 (0.59–0.86)
Contralateral vaulting	0.71 (0.22–0.85)

Data are presented as median (from minimum to maximum) of the Spearman’s rank correlation coefficient values of all participants.

## Discussion

This study investigated the criterion validity of a novel gait analysis system for detecting abnormal gait patterns in individuals with hemiparetic stroke during robot-assisted gait training in healthy adults, with reference to index values calculated by a marker-based motion capture system. The strength of the correlation between the index values of the two methods in each abnormal gait pattern was determined to be moderate to very high. Therefore, the index values of abnormal gait patterns detected by the proposed novel gait analysis system in the robot have criterion validity.

The WW-2000 is a robot-assisted gait training system that utilizes a treadmill. A treadmill is suitable for skeletal tracking in a markerless motion capture system because it acquires repetitive gait cycle data in a limited space (Clark et al., 2019). Previous studies using a typical markerless motion capture system, Microsoft Kinect, have reported that the system was able to match time and distance factors and hip and knee joint angles (Eltoukhy et al., 2017) and could accurately track angular changes in the trunk (Macpherson et al., 2016), compared with a reference marker-based motion capture system. On the other hand, several studies have reported that, although trends in joint motion of the hip and knee joints can be tracked in the markerless motion capture system, there are significant errors in the degree of angulation (Pfister et al., 2014; Xu et al., 2015). In addition, a markerless motion capture system using depth information in gait analysis showed a discrepancy in the timing of acquiring events between gait cycles because the measurement error increases when a distant object is captured [30]. Furthermore, estimating abnormal hemiparetic gait patterns is difficult with a markerless motion capture system, including the VisionPose^®^ used in this study, which estimates the joint positions based on a model developed from the postures and movements exhibited by healthy individuals in their daily lives (NEXT-SYSTEM Co., Ltd, 2022). However, the index values calculated by the proposed gait analysis system in this study showed good correlation with those calculated by an existing marker-based motion capture system. The reason for this might be attributed to the method in which the index values of the proposed gait analysis system were calculated (Nakashima et al., 2020). We calculated the index values of the proposed gait analysis system using not only the information obtained by the 3D sensor but also the information detected by multiple sensors, including the inertial, knee angle, and load sensors, which could detect the gait cycle more accurately (Nakashima et al., 2020). Regarding the inertial sensor, gait analysis methods that use wearable sensors have been widely introduced (Tao et al., 2012). When such inertial sensors are used, measurement accuracy is sometimes reduced by the drift errors of the inertial sensor in the yaw angle (Lopez-Meyer et al., 2011; Cardarelli et al., 2019, 2020) and magnetic disturbances when using geomagnetic sensors to estimate gait characteristics (de Vries et al., 2009). Various algorithms and combinations of sensors are used to overcome this low measurement accuracy (Lopez-Meyer et al., 2011; Cardarelli et al., 2019, 2020). The inertial sensor used in our proposed gait analysis system enables reliable acquisition of pitch and roll angles using a hybrid algorithm based on signals from a gyro sensor and an accelerometer (TAMAGAWA SEIKI Co., Ltd., 2022). To calculate abnormal gait patterns index values using the tilt of the robot in the novel gait analysis system, pitch and roll angles were used and changes in the tilt during task performance were calculated from the standing posture recorded during calibration. Therefore, we believe that the tilt of the robot calculated by the novel gait analysis system was not affected by the drift problem and magnetic disturbances caused by the motors of the treadmill or the robot and that reliable index values for abnormal gait patterns were calculated.

One strength of this study was the inclusion of participants of different heights (range; 155 cm–182 cm). Within this height range, the proposed gait analysis system could detect index values of abnormal gait patterns with a high correlation to those determined through an existing marker-based motion capture system. The viewing range of the 3D sensor used in this study was 72° horizontally, 52° vertically, and 90° diagonally (TEKWIND Co., Ltd., 2022). The 3D sensor was placed 0.6 m above the treadmill surface, and the distance between the sensor and participant was 1.2 m. The sensor was placed such that the horizontal direction of the viewing range was vertically from the ground. It was theoretically possible to capture the gait patterns of subjects up to approximately 190 cm in height, and the study results were in agreement with these expectations. However, if the participant’s walking speed is not stable and the walking position moves forward, the participant may move out of the sensor’s viewing area. Thus, it may be necessary to closely examine the participant’s walking position with respect to the sensor when using this markerless motion capture system in a clinical setting.

This study has several limitations. First, abnormal hemiparetic gait patterns were simulated by healthy adults due to difficulty in recruiting individuals with hemiparetic stroke whose gait abnormalities ranged in severity. However, the range of simulated abnormal gait pattern index values reported in this study covers the range in individuals with hemiparetic stroke reported in previous studies (Itoh et al., 2012; Matsuda et al., 2016; Tanikawa et al., 2016, 2021; Hishikawa et al., 2018). Therefore, we believe that the method used in this study, in which healthy adults simulated abnormal gait patterns, was appropriate. Second, we did not determine whether our system was able to detect the parameters of the gait patterns in fast motion as the sensors used had a sampling frequency of 30 Hz. However, individuals with hemiparetic stroke are expected to walk at a slower speed due to the severity of their gait disorder.

## Conclusion

The index values of abnormal gait patterns detected by the novel gait analysis system had a high correlation with those detected by a marker-based motion analysis system during the robot-assisted gait training. Thus, the proposed system showed criterion validity. This novel gait analysis system can help assess gait characteristics in individuals with hemiparetic stroke during robot-assisted gait training and provide individually tailored gait training based on these assessments.

## Data availability statement

The raw data supporting the conclusions of this article will be made available by the authors, without undue reservation.

## Ethics statement

The studies involving human participants were reviewed and approved by the Institutional Review Board of the Fujita Health University, Japan (approval number: CR19-027). The patients/participants provided their written informed consent to participate in this study.

## Author contributions

DI collected and analyzed the data. DI, SH, MM, and YO interpreted the data. DI and SH wrote the manuscript. All authors designed the study, reviewed the manuscript, read, and approved the final manuscript.

## Abbreviations

3D: three dimensional.
